# Carbapenem-Resistant Enterobacteriaceae (CRE) in Children with Cancer: The Impact of Rapid Diagnostics and Targeted Colonization Strategies on Improving Outcomes

**DOI:** 10.3390/microorganisms13071627

**Published:** 2025-07-10

**Authors:** Youssef Madney, Sally Mahfouz, Ahmed Bayoumi, Omayma Hassanain, Omneya Hassanain, Ahmed A. Sayed, Deena Jalal, Maryam Lotfi, May Tolba, Ghada A. Ziad, Mervat Elanany, Mohamed Hashem, Gehad Taha, Lobna Shalaby, Alaa Elhaddad

**Affiliations:** 1 Pediatric Oncology Department, Children’s Cancer Hospital Egypt (CCHE-57357), Cairo 11311, Egypt; sally.mahfouz@57357.org (S.M.); lobna.shalaby@57357.org (L.S.); alaa.hadad@57357.org (A.E.); 2 Pediatric Oncology Department, National Cancer Institute, Cairo University, Cairo 12613, Egypt; ahmed.kamal@y7mail.com; 3 Research Department, Children’s Cancer Hospital Egypt (CCHE-57357), Cairo 11311, Egypt; omayma.hassanain@57357.org (O.H.); omneya.hassanain@57357.org (O.H.); mohamed.diaaeldin@57357.org (M.H.); 4 Genomic and Metagenomics Research Program, Department of Basic Research, Children’s Cancer Hospital Egypt (CCHE-57357), Cairo 11311, Egypt; ahmad.sayed@57357.org (A.A.S.); deena.jalal@57357.org (D.J.); mariam.lotfi@57357.org (M.L.); 5 Department of Biochemistry, Faculty of Science, Ain Shams University, Cairo 11566, Egypt; 6 Department of Pharmaceutical Services and Sciences, Children’s Cancer Hospital Egypt (CCHE-57357), Cairo 11311, Egypt; may.m.hassan.a@gmail.com; 7 Microbiology Unit, Children’s Cancer Hospital Egypt (CCHE-57357), Cairo 11311, Egypt; ghada.aly@57357.org (G.A.Z.); mervat.elanany@57357.org (M.E.); 8 Clinical Pathology Department, Faculty of Medicine, Cairo University, Cairo 12613, Egypt; 9 Department of Surgical Oncology, Children’s Cancer Hospital Egypt (CCHE-57357), Cairo 11311, Egypt; gehad.taha@57357.org

**Keywords:** carbapenem-resistant Enterobacteriaceae (CRE), children, cancer, rapid diagnostics

## Abstract

Carbapenem-resistant Enterobacteriaceae (CRE) pose an emerging threat, with a high mortality rate among children with cancer. This study aimed to evaluate the impact of routine rectal swab surveillance and rapid PCR-based detection of carbapenemase genes to facilitate the early initiation of appropriate treatment and assess its effects on outcomes. The study compared two groups of pediatric cancer patients with CRE bloodstream infections: a retrospective cohort of 254 patients from 2013 to 2017, and a prospective cohort of 186 patients from 2020 to 2022, following the implementation of these tools. A rapid diagnostic test in the prospective cohort resulted in the early initiation of proper antibiotics in 85% (165/186) of patients, compared to only 58% (147/254) in the retrospective group. This led to a decrease in the need for ICU admission related to sepsis from CRE and a significant reduction in the 30-day mortality rate (16% vs. 30%, *p* ≤ 0.01). Genotypic profiling revealed that class B carbapenemases were the most prevalent (69%), with the NDM type being identified in 67% of patients. OXA-48 and KPC enzymes were detected in 59% and 4% of patients, respectively. Multivariate analysis revealed that patients having Klebsiella pneumoniae, NDM genotype carbapenemases, presence of pneumonia, and septic shock requiring ICU admission were predictors of poor outcomes. Rapid diagnostics and targeted colonization lead to the appropriate use of targeted antibiotics, resulting in improved patient outcomes. Understanding carbapenemase-producing microorganisms and administering newer antibiotics may further reduce mortality and enhance treatment strategies for high-risk patients.

## 1. Introduction

Pediatric cancer patients with carbapenem-resistant Enterobacteriaceae (CRE) infections had poor clinical outcomes with increased morbidity and mortality [1,2]. *Escherichia coli* (*E. coli*) and *Klebsiella pneumoniae* are the most isolated pathogens among those patients [3,4,5]. According to the Clinical and Laboratory Standards Institute (CLSI), an Enterobacteriaceae isolate is considered CRE if it has a minimum inhibitory concentration (MIC) ≥ 2 μg/mL for ertapenem or ≥4 μg/mL for meropenem, imipenem, or doripenem, or documentation that the isolate produces a carbapenemase [6].

Carbapenemases are a type of β-lactamase that can hydrolyze carbapenem antibiotics, rendering them ineffective [7]. The Ambler classification categorizes carbapenemases into four classes: Class A includes KPC (Klebsiella pneumoniae carbapenemase), Class B consists of metallo-β-lactamases (MBLs) such as NDM (New Delhi metallo-beta-lactamase), Class C includes AmpC β-lactamases that can hydrolyze cephalosporins and certain carbapenems, and Class D encompasses oxacillinases (OXA-type), which also hydrolyze carbapenems but differ in their mechanism [8,9,10]. The production of carbapenemases, such as K. pneumoniae carbapenemase (KPC), oxacillinase (OXA)-48-like enzymes (OXA-48), and New Delhi metallo-β-lactamases (NDM), is the primary mechanism of resistance [8]. The distribution of carbapenemases varies geographically; KPC enzymes are prevalent in North America and Europe, but are less reported in the Middle East, whereas metallo-β-lactamases (MBLs) are more prevalent [11]. Ambler class D oxacillinases (such as OXA-48) are increasingly being identified in Europe and occasionally in the United States [12].

Patients with hematological malignancies are at risk for CRE bacteremia due to gastrointestinal mucositis, underlying malignancies, chemotherapy-induced neutropenia, and prolonged hospital stays [13,14]. Carbapenem-resistant Enterobacteriaceae (CRE) colonization in cancer patients significantly increases the risk of bloodstream infections, especially during mucosal barrier damage following chemotherapy, when the compromised mucosa allows bacterial leakage into the bloodstream [15,16]. Multiple clinical studies have demonstrated that CRE colonization is associated with a higher risk of CRE infection and mortality in patients [4,17]. Many guidelines recommend actively screening for CRE and implementing effective infection prevention and control strategies to prevent its spread [18,19]. Identifying the risk factors that contribute to the transition from CRE colonization to infection will reduce the incidence of CRE infections and mortality [19]. The mortality rate among neutropenic patients with CRE bacteremia was reported to be around 60% [13,14]. Recent guidelines recommend new antibiotics; however, the unavailability of these medications is frequent in countries with limited resources [20,21].

The previous study at our center reported a mortality rate of about 30% among 254 pediatric cancer patients with (CRE) bloodstream infections (BSIs) between 2013 and 2017 [3]. In our retrospective study, surveillance for CRE colonization screening was not routinely conducted. However, 30% of patients with CRE BSIs were found to be colonized with CRE, as identified by either rectal swab or stool culture. A key finding in the data analysis indicates that delaying the initiation of adequate antibiotic treatment for more than 48 h, until the results of the routine blood culture diagnostic test were available, was associated with a high mortality rate. Based on the data reported in this study, rapid PCR diagnostic tests were adopted to ensure the timely administration of effective therapies, and routine rectal swabs were taken from patients during admission for chemotherapy in our prospective cohort. If a patient exhibited CRE colonization, strict infection control measures and isolation were implemented. Also, empirical antibiotic treatment targeting CRE colonization was initiated once the patient developed febrile neutropenia and was continued until the blood culture results were available. Rapid de-escalation was performed if the culture was negative for CRE-BSI.

## 2. Materials and Methods

### 2.1. Study Design

The study applied a mixed-methods approach, comparing a prospective cohort with a retrospective group, to evaluate the effectiveness of routine rectal swab surveillance and rapid diagnostics. The prospective group consisted of 186 children with cancer who had CRE bloodstream infections (BSIs) during the studied period (2020–2022) at a Children’s Cancer Hospital in Egypt (CCHE). Among this study group, we applied routine screening rectal swabs for high-risk patients (acute leukemia and hematopoietic stem cell transplant patients), started active treatment for those colonized by CRE, and introduced a rapid PCR-based method for carbapenemase gene detection, with available results within one hour, allowing for the rapid initiation of targeted antibiotics in <48 h. The retrospective study included 254 CRE-BSIs among pediatric cancer patients (2013–2017). Resistance to carbapenems was defined using the Clinical and Laboratory Standards Institute (CLSI, 2022) criteria [6]. Clinical characteristics, microbiological data with genotypic resistance profile, antimicrobial treatment, and clinical outcomes were analyzed. The primary endpoint was 30-day mortality after the first positive culture isolate of CRE. This study was approved by the Institutional Review Board (IRB) for the ethical committee. Informed consent was obtained from the patient’s legal guardians prior to any diagnostic or therapeutic intervention.

### 2.2. Fever and Neutropenia Protocol Guidelines

The Infectious Diseases Society of America (IDSA) guidelines for managing febrile neutropenia were adopted [22]. Antifungal prophylaxis (Voriconazole 9 mg/kg/dose every 12 h) with antibacterial levofloxacin (10 mg/kg/d for patients more than 5 years with maximum 750 mg/day and 10 mg/kg/dose twice daily if <5 years with maximum 500 mg/day) was administered to patients with AML. For initial empirical antimicrobial therapy, a combination of carbapenem and aminoglycosides was used in high-risk febrile neutropenic patients, based on the institutional antibiogram, which indicated a rising incidence of Extended-Spectrum β-lactamase (ESBL)-producing Gram-negative bacteremia.

### 2.3. Treatment Guidelines for CRE-BSI

Patients with carbapenem-resistant Enterobacterales bloodstream infections (CRE-BSIs) were treated based on the minimum inhibitory concentration (MIC) of carbapenem. For patients with CRE and MIC < 8 µg/mL, the primary treatment involved a high-dose continuous infusion of carbapenem at 40 mg/kg over 3 h, combined with a second active agent. If the isolate was susceptible, the second agent would be an aminoglycoside; if resistant, colistin would be used. In patients with CRE and an MIC of >8 µg/mL, colistin was the primary treatment. In these cases, if the isolate was susceptible to aminoglycosides, this agent was added as a second treatment; if it was resistant, tigecycline was used instead. In the prospective study, according to the genotypic resistance profile, if KPC or OXA-48 carbapenemases were detected, ceftazidime-avibactam (CAZ-AVI) was added as a monotherapy or combined with aztreonam if NDM carbapenemases were detected. These antibiotics were reserved as salvage therapy for CRE after the microbiological failure of polymyxins for more than 5–7 days. The duration of antimicrobial therapy for CRE-BSI was 10–14 days. For patients with a documented site of infection, such as gastrointestinal infection or pneumonia, the duration of treatment was adjusted according to the recovery of neutrophil counts and clinical, radiological, and microbiological responses.

### 2.4. Microbiological Detection of CRE

Pediatric blood culture bottles (BACTECTM PedsPlusTM/F/F, BD Diagnostics & BacT/ALERT^®^PF Plus, BD Diagnostics, Sparks, MD, USA) inoculated with patients’ blood were placed into the corresponding blood culture system. Positive blood culture broths were sub-cultured onto Columbia blood agar and MacConkey No. 2 agar plates (Oxoid™, Thermo Scientific, Waltham, MA, USA) and incubated at 37 °C. The isolated colonies were identified using the MALDI-TOF Vitek MS IVD system (BioMérieux; Marcy l’Etoile, France). Xpert^®^ Carba-R assay (Cepheid, Sunnyvale, CA, USA) and real-time PCR were used to detect five carbapenemase gene families, including *bla*_IMP (IMP-1, 3, 6, 10, 25 & 30)_, all *bla_KPC_*, all *bla*_NDM_, *bla*_OXA-48_-like _(OXA-48, 162, 163, 181, 204, 232, 244, 245 & 47)_*,* and all *bla*_VIM_, which are associated with carbapenem nonsusceptibility in Gram-negative bacterial infections. This assay was performed on the GeneXpert instrument, and results are available within one hour [23,24]. The Automated Vitek 2 Compact (BioMérieux SA, Marcy l’Étoile, France) was used to test isolates on agar plates for antibiotic susceptibility, as recommended by the manufacturer, with the results interpreted according to the guidelines of the Clinical and Laboratory Standards Institute (CLSI).

### 2.5. Definitions

Multidrug-Resistant (MDR) Bacteremia: Refers to bacteremia caused by pathogens that show resistance to one agent in three or more categories of antimicrobials [25]. Carbapenem-Resistant Enterobacteriaceae (CRE): Defined as Enterobacteriaceae resistant to carbapenem antibiotic, with a (MIC) > 4 mg/mL for imipenem, meropenem, or doripenem, or an ertapenem MIC > 2 mg/mL [6]. Sepsis or Septic Shock: Conducted according to the definitions established by the International Consensus Conference on Pediatric Sepsis [26]. The primary outcome was clinical success 14 days after CRE infection. Clinical success was defined as the patient being alive, achieving hemodynamic stability (systolic blood pressure > 90 mm Hg without vasopressor support), and demonstrating a stable or improved ratio of partial pressure of arterial oxygen to expired oxygen in pneumonia cases. Patients not meeting these criteria were classified as clinical failures.

### 2.6. Statistical Analysis

Statistical analysis was performed by using IBM SPSS^®^ Statistics version 22. Numerical data were presented as medians with ranges or means with standard deviations. Qualitative data were expressed as percentages and frequencies, while quantitative data were tested for normality using the Kolmogorov–Smirnov and Shapiro–Wilk tests. Risk estimation was expressed as odds ratios (ORs) with 95% confidence intervals (CIs). Logistic regression was performed to identify which candidate variables best discriminate against the resistant species, and then multivariable logistic regression analysis was used to examine all risk factors together. Multivariate logistic regression analysis was used to identify significant factors affecting outcomes (30-day survival) based on univariate analysis. A *p*-value < 0.05 was considered statistically significant.

## 3. Results

### 3.1. Clinical Characteristics of CRE Patients

The retrospective study included 254 pediatric cancer patients with CRE-BSI from 2013 to 2017, with a median age of 6 years and a predominance of male patients (58%; 124/254). More than 70% of the patients had hematological malignancies, with AML being the most prevalent (54.0%). Of the 254 patients, 130 (51.0%) had refractory or relapsed disease. The prospective cohort involved 186 pediatric cancer patients with CRE BSI from 2020 to 2022, with a slightly older median age of 9 years and a similar gender distribution (59.0%; 110/186 male). This cohort also had a high prevalence of hematological malignancies (80.0%; 124/150), with AML remaining the most common (47.0%). Furthermore, 33.0% (61/186) of these patients suffered from refractory or relapsed disease. Hospitalization patterns showed that in both cohorts, CRE-BSI was mainly acquired in medical wards (85–90%), with approximately 10–15% occurring in the Intensive Care Unit (ICU) during the bacteremia episode (Table 1).

Our analysis showed that both cohorts had similar clinical features for acquiring CRE infection. Profound neutropenia lasting more than 7 days was observed in 80% of the cases. Also, the majority of patients had prior carbapenem exposure within the last 90 days (80–90%). Other features included quinolone prophylaxis (30.0%), a central venous catheter (25–45%), and a previous history of ICU admission (around 25%). Thirty-five percent of patients (90/254) were colonized with carbapenem-resistant Enterobacteriaceae (CRE), a key finding from the retrospective study. Based on this, the following prospective study included routine CRE screening for all high-risk patients, especially patients with AML and those receiving salvage therapy for relapsed leukemia. This screening revealed that 36% of these patients were colonized with CRE, and a similar percentage, 35% (67 of 186), had colonization with extended-spectrum beta-lactamase (ESBL)-producing organisms. Moreover, around 50.0% of the patients in both cohorts had documented sites of infection, together with bloodstream infections. Overall, 30% had pneumonia, typhlitis was reported in 20.0%, and urinary tract infection in 10.0%, while skin and soft tissue infection was observed in 20 to 40% of the total patients (Table 1).

### 3.2. Isolated Pathogens and Resistance Patterns

In both studies, a similar pattern in bacterial isolation was detected. The most common isolated bacteria were *E. coli* and *Klebsiella pneumoniae*. These findings highlight the consistency in clinical features and bacterial profiles across both studies. The current data analysis revealed differences in the resistance pattern between the two studies. In the retrospective study, carbapenem resistance was observed in all patients, with 45% exhibiting a minimum inhibitory concentration (MIC) of less than 8 µg/mL and 55% showing an MIC > 8 µg/mL. Resistance to aminoglycosides was noted in 46.5% of cases, while 87% were resistant to quinolones. Colistin resistance was identified in 1% of isolates (two isolates), and no resistance was observed against tigecycline. In contrast, the prospective study found that 100% of patients exhibited carbapenem resistance, with an MIC greater than 8 µg/mL. Aminoglycoside resistance was present in 30% of patients, and 79% showed resistance to quinolones. Colistin resistance was reported in 5% (nine isolates), while tigecycline resistance remained absent. These findings highlighted an alarming increase in the incidence of colistin resistance, a growing challenge in managing CRE infections.

### 3.3. Genotypic Profile

Genotypic profiling of CRE-BSI revealed that class B carbapenemases were the most prevalent, accounting for 69.0% of cases. Among these, New Delhi metallo-beta-lactamase (NDM) was identified in 126 out of 186 patients (67.0%), while Verona integron-encoded metallo-beta-lactamase (VIM) was found in 8 patients (4.0%). OXA-48 carbapenemases were detected in 110 patients (59.0%), whereas KPC enzymes were reported in only 7 (4.0%) patients (Supplementary Table S1). Incorporating genotypic profiling in the prospective study had significant epidemiological and therapeutic implications. Notably, recent antibiotics such as ceftazidime-avibactam are ineffective against class B carbapenemases. Therefore, if these antibiotics are administered, they should be combined with aztreonam to enhance treatment efficacy.

### 3.4. Clinical Outcomes

In the retrospective study, 42% of the patients had a delay in starting adequate antibiotics against CRE (>48 h), and most of them experienced septic shock, necessitating admission to the intensive care unit (ICU) and inotropic support. The overall mortality rate was 55%, with a 30-day mortality rate of 30% (76/254) following the onset of bloodstream infections (BSI). On the other hand, applying the new rapid diagnostic test in the prospective cohort resulted in a marked reduction in initiating the appropriate antibiotics, with more than 85% of patients (165/186) receiving active antibiotic treatment within 48 h. This led to a decrease in the need for ICU admission related to sepsis from CRE. Also, there was a significant reduction in the 30-day mortality rate (16% vs. 30%, *p* ≤ 0.01) (Table 2). These findings highlight significant differences in treatment timing, outcomes, and mortality rates between the retrospective and prospective studies, underscoring the importance of timely and appropriate antibiotic therapy in managing CRE infections.

## 4. Discussion

Carbapenem-resistant Enterobacteriaceae (CRE) are among the leading causes of mortality among pediatric cancer patients. This directed our attention to collecting data from 2013 to 2017, analyzing the clinical features and outcomes of CRE-BSI, and developing a new strategic management plan. In our center, 254 children with cancer had CRE bacterial infections, with the majority (74%) having hematological malignancies. The most isolated pathogens were *E. coli* and *K. pneumoniae*. Key clinical features included severe, prolonged neutropenia, prior antibiotic exposure, steroid use, quinolone prophylaxis, ICU admission, and central venous catheter use. CRE colonization occurred in one third of patients. Previously, identifying pathogens and their resistance profiles could take days, leading to delayed appropriate treatment and the unnecessary use of broad-spectrum antibiotics, which contributed to the development of resistance. Poor survival outcomes were linked to septic shock, inadequate empirical therapy, and delayed adequate treatment (>48 h). The 30-day mortality rate was 30%, highlighting CRE as a significant emerging threat to pediatric cancer outcomes.

To tackle CRE-BSI-related mortality in pediatric cancer patients, implementing routine rectal swab surveillance with a targeted colonization treatment strategy and rapid diagnostics is crucial. Given that prior CRE colonization is a critical risk factor for subsequent bloodstream infections, and delayed initiation of active treatment for more than 48 h after culture was associated with higher mortality, then improved surveillance, preventive strategies, and targeted treatment can reduce this risk, improve patient outcomes, and combat antimicrobial resistance.

A two-step approach was proposed. First, we screened high-risk patients using rectal swabs to inform preventative measures and guide initial antibiotic treatment during febrile neutropenia. The mechanisms linking colonization to infection, supported by epidemiological evidence, highlight the importance of addressing colonization in clinical settings [27,28]. As a result, we implemented rectal swab screening for high-risk leukemia patients, revealing that over two thirds were colonized with either CRE or ESBL. Applying infection control measures, including hand hygiene, and understanding the relationship between colonization and infection guided us in using colonization-guided empirical antibiotics for suspected infections in at-risk patients. Second, we utilized rapid PCR testing to facilitate the rapid identification of Gram-negative bacteria and their resistance mechanisms within hours [29]. This enabled prompt, targeted therapy within 48 h, leading to improved patient outcomes (a reduction in mortality rates from 30% to 15%) and minimizing unnecessary broad-spectrum antibiotic use, which ultimately reduced the selective pressure that fosters further resistance.

Understanding the resistance patterns within a specific healthcare facility is essential for effectively tailoring antibiotic guidelines [20]. In our cohort, class B carbapenemase (69%) was the most common, with NDM detected in 67% of cases, followed by OXA-48 (59%) and KPC (4%). Carbapenemases, enzymes that cause antibiotic resistance in Gram-negative bacteria, hydrolyze last-resort carbapenems, leading to increased infections, mortality, and hospital stays. Identifying the prevalent carbapenemase-producing organism (CPO) strains and their resistance mechanisms will assist in formulating targeted empirical treatment protocols. New antibiotics should align with this genetic profile: Ceftazidime-avibactam effectively treats OXA-48 and KPC, while combining Ceftazidime-avibactam with Aztreonam targets NDM-producing pathogens [20].

Novel antimicrobial agents, including cefiderocol, meropenem-vaborbactam, imipenem-relebactam, and aztreonam-avibactam, provide effective options for treating carbapenem-resistant Enterobacteriaceae (CRE) infections [10]. The Infectious Diseases Society of America (IDSA) and the European Congress of Clinical Microbiology and Infectious Diseases (ECCMID) recommend these agents, with the IDSA advocating for their use based on efficacy [20]. ECCMID emphasizes a tailored approach, suggesting meropenem-vaborbactam or ceftazidime-avibactam for severe cases, while conditionally recommending cefiderocol for highly resistant strains [21].

Ceftazidime-avibactam (CAZ-AVI), a novel beta-lactam/beta-lactamase inhibitor, plays a crucial role in treating infections caused by difficult-to-treat CRE, particularly those involving OXA-48 and KPCs [30]. A meta-analysis reported a high cure rate (68.4%) and a lower 30-day mortality rate (39.5%) with CAZ-AVI, reflecting its effectiveness compared to traditional therapies. Thus, CAZ-AVI significantly impacts the management of multidrug-resistant infections, improves survival, and provides better control of severe CRE-related infections [30,31]. In our protocol, CAZ-AVI, either as monotherapy or in combination with aztreonam, is reserved as a salvage therapy for CRE following the microbiological failure of polymyxins. Moving forward, our next antimicrobial stewardship goals will focus on identifying high-risk features that can predict mortality. The early use of CAZ-AVI as a first-line treatment could improve outcomes and reduce mortality rates to below 15%, as achieved with our current treatment strategies.

One of the primary goals of the current study is to identify predictors of mortality. Multivariate analysis of both retrospective and prospective studies identified significant predictors associated with poor outcomes, such as the isolation of *Klebsiella pneumoniae*, associated typhlitis or pneumonia, carbapenem resistance with a MIC > 8 µg/mL, or CRE patients who developed septic shock requiring ICU admission, as well as NDM genotype carbapenemases. Based on these predictors, we identified a high-risk mortality group among CRE-BSI patients who may benefit from the early initiation of CAZ-AVI rather than as a salvage option to reduce mortality and improve survival.

## 5. Conclusions

CRE-BSI in countries with limited resources remains a barrier to improving outcomes for children with cancer. Implementing routine colonization screening for high-risk patient groups, utilizing targeted colonization strategies, and employing a rapid diagnostic test (GeneXpert) has been shown to enhance outcomes and reduce mortality by facilitating the timely and appropriate initiation of targeted antibiotics. Understanding the prevalent carbapenemase-producing organisms (CPO) and their resistance mechanisms, along with identifying predictors of mortality, can strengthen effective treatment strategies.

## Figures and Tables

**Table 1:** The main clinical features of pediatric cancer patients with CRE-BSI.

	Retrospective Study	Prospective Study
**Study period**	2013–2017	2020–2022
**Number of patients with CRE-BSI**	254	186
**Age (median)**	6 years	9 years
**Gender** - Male - Female	165 (58%) 89 (42%)	110 (59.1%) 76 (40.9%)
**Primary malignancy**		
- **Hematological malignancies** - Acute myeloid leukemia - Acute lymphoblastic leukemia - Others - **Solid tumors**	**188 (74%)** 101 (40%) 59 (23%) 28 (11%) **66 (26%)**	**148 (80%)** 87 (47%) 53 (29%) 8 (4%) **38 (20%)**
**Disease status**		
- In remission - Refractory disease	124 (49%) 130 (51%)	125 (67%) 61 (33%)
**Acquisition of BSI**		
- Medical word - Intensive Care Unit (ICU)	220 (87%) 34 (13%)	166 (90%) 20 (10%)
**Main clinical features**		
- Prolonged neutropenia > 7 days	213 (84%)	155 (83%)
- Previous carbapenem exposure	203 (80%)	154 (83%)
- Antibiotic prophylaxis	77 (30%)	66 (35%)
- Central venous catheter use	61 (24%)	88 (47%)
- ICU admission within 90 days	72 (28%)	41 (22%)
- Colonization with CRE	90 (35%)	67 (36%)
**CRE Pathogen**		
- *E. coli*	151 (59%)	115 (62%)
- *Klebsiella pneumoniae*	94 (37%)	64 (34%)
**Source of BSI**		
**- Bacteremia (BSI only)**	112 (44%)	85 (45%)
**BSI with associated documented site infection**	142 (56%)	100 (55%)
- Pneumonia	68 (26.8%)	68 (36%)
- Skin and soft tissue	50 (19.7%)	90 (48%)
- Typhlitis	47 (18.5)	32 (17%)
- Urinary tract infection	15 (6%)	21 (11%)

**Table 2:** Impact of implementation of antimicrobial stewardship program on clinical outcome of pediatric cancer patients with CRE-BSI.

Clinical Outcome	Retrospective Study	Prospective Study	*p* Value
**Patients with CRE-BSI (N)**	**254**	**186**	
**Time to start active antibiotics**			
- Less than 48 h	**147 (58%)**	**165 (89%)**	0.001
- More than 48 h	**107 (42%)**	**21 (11%)**	
**Septic shock and ICU admission**			
- ICU admission	**90 (35.8%)**	**44 (24%)**	0.03
- Inotropic support need	**72 (28.3%)**	**35 (19%)**	0.04
**30-Day mortality**	**76 (30%)**	**31 (16%)**	0.01

## Data Availability

The original contributions presented in this study are included in the article/Supplementary Materials. Further inquiries can be directed to the corresponding author.

## Supplementary Materials

The following supporting information can be downloaded at https://www.mdpi.com/article/10.3390/microorganisms13071627/s1, Table S1: Genotypic profile of CRE among 186 pediatric cancer patients.
