# Supplementary material for: Carbapenem-Resistant Enterobacteriaceae (CRE) in Children with Cancer: The Impact of Rapid Diagnostics and Targeted Colonization Strategies on Improving Outcomes
Source: Microorganisms. 2025 Jul 10;13(7):1627. doi: 10.3390/microorganisms13071627 (PMC12300897; doi:10.3390/microorganisms13071627)
Supplement: Supplementary file 1 [file microorganisms-13-01627-s001.zip › microorganisms-3690887-supplementary.pdf]

Supp Table S1: Genotypic profile of CRE among 186 pediatric cancer patients

Genotypic profile of CRE	N = 186 (100%)
Class A (KPC)	7/186 (4%)
Class B<br>- NDM<br>- VIM	134/186(72%)<br>126/186(67%)<br>8/186(4%)
Class D (OXA-48)	110/186(59%)
Amp C	3/186 (2%)
